# Metabolic Engineering of *Enterobacter aerogenes* for Improved 2,3-Butanediol Production by Manipulating NADH Levels and Overexpressing the Small RNA RyhB

**DOI:** 10.3389/fmicb.2021.754306

**Published:** 2021-10-08

**Authors:** Yan Wu, Wanying Chu, Jiayao Yang, Yudong Xu, Qi Shen, Haoning Yang, Fangxu Xu, Yefei Liu, Ping Lu, Ke Jiang, Hongxin Zhao

**Affiliations:** ^1^Zhejiang Province Key Laboratory of Plant Secondary Metabolism and Regulation, College of Life Sciences and Medicine, Zhejiang Sci-Tech University, Hangzhou, China; ^2^Key Laboratory of Urban Agriculture by Ministry of Agriculture of China, School of Agriculture and Biology, Shanghai Jiao Tong University, Shanghai, China; ^3^Department of Bioengineering, Liaoning Technical University, Fuxin, China; ^4^Experimental Teaching Center, College of Life Science, Shenyang Normal University, Shenyang, China

**Keywords:** *Enterobacter aerogenes*, NADH dehydrogenase, lactate dehydrogenase, small RNA RyhB, 2,3-butanediol

## Abstract

Biotechnological production of 2,3-butanediol (2,3-BD), a versatile platform bio-chemical and a potential biofuel, is limited due to by-product toxicity. In this study, we aimed to redirect the metabolic flux toward 2,3-BD in *Enterobacter aerogenes* (*E. aerogenes*) by increasing the intracellular NADH pool. Increasing the NADH/NAD^+^ ratio by knocking out the NADH dehydrogenase genes (*nuoC*/*nuoD*) enhanced 2,3-BD production by up to 67% compared with wild-type *E. aerogenes*. When lactate dehydrogenase (*ldh*) was knocked out, the yield of 2,3-BD was increased by 71.2% compared to the wild type. Metabolic flux analysis revealed that upregulated expression of the sRNA RyhB led to a noteworthy shift in metabolism. The 2,3-BD titer of the best mutant Ea-2 was almost seven times higher than that of the parent strain in a 5-L fermenter. In this study, an effective metabolic engineering strategy for improved 2,3-BD production was implemented by increasing the NADH/NAD^+^ ratio and blocking competing pathways.

## Introduction

The biotechnological production of bulk chemicals is partially driven by the wish to reduce the reliance on fossil fuels because of limited reserves and increasing environmental concerns. The small molecule 2,3-BD is listed by the United States Department of Energy as a platform chemical with great potential for industrial applications (Song et al., 2019). The production of 2,3-BD by bacteria such as *Escherichia* sp. (Park et al., 2020), *Bacillus* sp. (Petrova et al., 2020), *Klebsiella* sp. (Sharma et al., 2018), and *Enterobacter* sp. (Thapa et al., 2019) has been investigated in depth in the last few years on account of its potential applications in the chemical industry for the production of polymers and solvents. As a versatile chemical feedstock and liquid fuel, 2,3-BD is extensively applied in the food, chemical, pharmaceutical, petrochemical, and aerospace industries (Ge et al., 2016). The development of biorefinery technology was further boosted due to concerns surrounding high energy costs and environmental problems (Ragauskas et al., 2006; Haveren et al., 2008). Most microbiological methods for the production of 2,3-BD rely on the biotransformation of expensive glucose. However, the utilization rate of glucose is still low, resulting in a pressing need for more efficient biological production methods for 2,3-BD.

*Enterobacter aerogenes* (syn. *Klebsiella aerogenes*) is a facultatively anaerobic Gram-negative bacterium that naturally produces 2,3-BD during fermentation at low O_2_ concentrations. Due to its role in maintaining the intracellular redox balance of the pyridine nucleotide pool during glycolysis and biosynthesis, 2,3-BD was regarded as a classical end-product of anaerobic fermentation (Converti et al., 2003). The conversion of acetoin by 2,3-BD dehydrogenase regenerates NADH for continued glycolysis, and this reaction is increased to maintain the NAD^+^/NADH balance when there is a lack of oxygen (Blomqvist et al., 1993). Because NADH from glycolysis is regenerated *via* respiration under aerobic conditions, the switch from biomass synthesis to production of mixed acids and 2,3-BD is critical for maintaining the redox balance (Hung et al., 2011).

Many studies have demonstrated that small RNAs play critical roles in the metabolic regulation of both eukaryotes and bacteria (Beauchene et al., 2015; Machner and Storz, 2016). The small noncoding RNA RyhB undergoes base pairing with mRNAs to suppress gene expression (Massé et al., 2003). RyhB is related to a variety of crucial cellular functions such as iron homeostasis, antioxidant defenses, and TCA cycle activity in many bacteria, including *Klebsiella pneumoniae* and *Escherichia coli* (*E. coli*; Massé et al., 2003; Huang et al., 2012; Beauchene et al., 2015). In *E. coli*, formate dehydrogenase is downregulated by RyhB (Massé et al., 2005), suggesting that RyhB might regulate 2,3-BD production in *E. aerogenes* by suppressing formate production. Moreover, RyhB also influences the expression of NADH dehydrogenase I (Massé et al., 2005), which may influence the NAD^+^/NADH ratio during the production of 2,3-BD.

*Enterobacter aerogenes* can produce high titers of 2,3-BD from many different carbon sources, while producing different fermentation gases and soluble byproducts. Lactate is the main byproduct during the anaerobic fermentation process of *E. aerogenes* and blocking the lactate synthesis pathway can increase the yield of 2,3-BD due to their competition for NADH as a cofactor and pyruvate as a precursor. However, there are few reports on metabolic engineering in *E. aerogenes*. Nevertheless, a previous study improved hydrogen production in *E. aerogenes* by deleting a hydrogenase subunit and formate hydrogen lyase subunit, while also overexpressing a heterologous formate dehydrogenase and its positive regulator (Lu et al., 2009, 2016; Zhao et al., 2009). Similarly, Lu et al. (2010) investigated the effects of overexpressing formate hydrogenase and deleting lactate dehydrogenase (LDH) on hydrogen production. In a more recent study, CRISPR-Cas9 was used to knock out the *nuoCD* genes in the NADH dehydrogenase cluster of *E. aerogenes* IAM1183. The resulting double mutant Ea-1 (Δ*nuoC/*Δ*nuoD*) showed improved hydrogen production (Wu et al., 2017).

In this study, the gene *ldh* encoding LDH was deleted using the λ-Red recombination system to reduce byproduct accumulation, generating Ea-2 (*ΔnuoC*/*ΔnuoD*/*∆ldh*). To regulate the 2,3-BD production of IAM1183 *via* the small RNA RyhB, it was overexpressed in both strains Ea-1 and Ea-2, respectively, resulting in Ea-3 and Ea-4. We constructed a metabolic network model to quantify the metabolic fluxes of IAM1183 and its mutants and thereby analyze the changes in the metabolic pattern of the mutants. The best mutant exhibited a high 2,3-BD titer in both shake-flasks and batch fermentation, indicating its potential for scaled-up 2,3-BD production.

## Materials and Methods

### Strains, Plasmids, and Reagents

Table 1 lists the primers, plasmids, and strains used in this work. The Green Industry Biotechnology Laboratory of Tsinghua University kindly provided the *E. aerogenes* wild-type strain IAM1183. *Escherichia coli* DH5α (TaKaRa Biotechnology, Dalian, China) was used for genetic engineering. Kits for the isolation of genomic DNA, DNA gel extraction, and plasmid DNA purification were from GenScript (GenScript USA Inc. United States). All DNA-modifying enzymes, restriction endonucleases, and DNA polymerase were bought from New England BioLabs (Beverly, MA, United States). Tryptone and yeast extract were purchased from Thermo Fisher Biochemicals (Beijing Ltd.). Unless specified otherwise, all other reagents used in this study were purchased from Sigma-Aldrich (St. Louis, MO, United States).

**Table 1:** Strains, plasmids, and primers used in the experiments.

Strains, plasmids, and primers	Genotype and relevant characteristics	Source or literature
Strains		
IAM1183	Wild type	IAM (Tokyo, Japan)
E.a-1	*E. aerogenes* IAM1183 *DnuoC*/*DnuoD*	Wu et al., 2017
E.a-2	*E. aerogenes* IAM1183 *DnuoC*/*DnuoD*/*Dldh*	This study
E.a-3	*E. aerogenes* IAM1183 *DnuoC*/*DnuoD*, harboring plasmid pKK102-ryhB-cm	Wu et al., 2017
E.a-4	*E. aerogenes* IAM1183 *DnuoC*/*DnuoD*/*Dldh*, harboring plasmid pKK102-ryhB-cm	This study
*E. coli* DH5a	*F-, φ 80dlacZ DM15*, Δ (*lacZYA -argF*) *U169*, *deoR*, *recA1*, *endA1*, *hsdR17* (*rK-, mK+*), *phoA*, *supE44*, *λ-, thi −1*, *gyrA96*, *relA1*	TaRaKa
Plasmids		
pKD46	*FRT flanked resistance cassette involved vector*, *oriRγ*, *Amp^r^*	TaRaKa
pKD46-cm	FRT flanked resistance cassette involved vector, oriRγ, *Cm^r^*	This study
pKD46-ldh	*ldh*-homologous arm FRT flanked resistance cassette involved vector, oriRγ, *Cm^r^*	This study
pCP20	FLP recombinase producing vector, *Amp^r^*, *Cm^r^*	TaRaKa
pMD18-T Vector	TA Cloning vector, *Amp^r^*	TaRaKa
FRT	Kan+, FRT site	TaRaKa
pKK102-ryhB-cm	RyhB, *Cm^r^* deriving pKK102-ryhB	This lab
Primers (5'–3')		
Primer-cm-F	TGCACGGTGCACAGTGCCAAGC TTGCATGCCT	This study
Primer-cm-R	CGGCATGACTCCCCGTCAGTATACACT CCGCTAGCGC	This study
Ea-ldh-F	*TTGTACGATTATTTTAAATATGCTACCGTGACGGTATAATC* *ACTGGAGAAAAGTCTT* AATTAACCCTCACTAAAGGGCG	This study
Ea-ldh-R	*CTGTGGGGATTATCTGAATGTGCTCCCCCCGGGAGAGGAG* *CACAAAAGGGAAAGGCA* TAATACGACTCACTATAGGGCTC	This study
ldh-Con-A	AATTAACCCTCACTAAAGGGCG	This study
ldh-Con-B	TAATACGACTCACTATAGGGCTC	This study
ldh-Con-C	CCGTGACGGTATAATCACTGGAG	This study
ldh-Con-D	ATCTTCGAAG AACAGGTCGC	This study

### Media and Culture Conditions

Luria–Bertani (LB) broth including (g/L) 10 tryptone, 5 yeast extract, and 10 NaCl was used to culture *E. coli* for cloning and cryopreservation. The cultivation medium consisted of several reagents including (g/L) 6.8 Na_2_HPO_4_, 3 KH_2_PO_4_, 0.75 KCl, 0.28 Na_2_SO_4_, 5.35 (NH_4_)_2_SO_4_, 34.2 ZnCl_2_, 0.26 MgSO_4_·7H_2_O, 0.42 citric acid, 10 Casamino acids, 5 yeast extract, and 0.3ml trace element solution containing (g/L) 2.7 FeCl_3_·6H_2_O, 10 0.85 CuCl_2_·2H_2_O, MnCl_2_·4H_2_O, and 0.31 H_3_BO_3_ (Jung et al., 2012) used to produce 2,3-BD. Glucose, sucrose, galactose, and fructose (40g/L) were used as carbon sources. The 50ml cultures in 250-ml Erlenmeyer flasks were closed using silicone stoppers and incubated micro-aerobically at 37°C and 250rpm. Kanamycin (50mg/ml), chloramphenicol (25mg/ml), or ampicillin (50mg/ml) was added to the medium, respectively, when necessary.

Fermentation experiments were performed in a 5-L bioreactor (Biostat A, Sartorius, Germany) with 3L of fermentation medium, at 250rpm and 37°C. The same medium was used for fermentation as the flask culture, and the carbon source was glucose. Chloramphenicol (50mg/ml) was supplied to promote plasmid retention. The fermentation was conducted at 37°C and 250rpm, with air supplied at 1vvm. The pH was maintained in the range from 6.5 to 7.0 by adding 5M NaOH (Jung et al., 2012).

### Genetic Manipulation

Table 1 lists all primers used in this study. The *ldh* gene, which encodes LDH, was knocked out in the Ea-1 strain (*E. aerogenes* IAM1183 *DnuoC*/*DnuoD*) using a published λ-Red recombination method (Datsenko and Wanner, 2000), resulting in strain Ea-2 (*E. aerogenes* IAM1183 *DnuoC*/*DnuoD*/*Dldh*). Genomic DNA was isolated from *E. aerogenes* IAM1183 according to a published method (Miller, 1972) or using a commercial kit. The QIAprep Spin Miniprep kit and GenScript DNA gel extraction kit were used for plasmid isolation and DNA purification, respectively. The 50μl PCR reactions contained: PCR reaction buffer (5μl), dNTPs (10mM each), primers (10pmol each), 1U of Pfu DNA polymerase or Taq polymerase, and 10–20ng of the DNA template were conducted with a Bio-Rad thermocycler (Hercules, CA, United States). The correct products were confirmed by sequencing (Life Technologies, Shanghai Invitrogen, China). The purified plasmid pKK102-ryhB-cm, which was used to overexpress the small RNA RyhB, was introduced into Ea-3 by electroporation, and the resulting strain was named Ea-4 (*E. aerogenes* IAM1183 *DnuoC*/*DnuoD*/*Dldh* [pKK102-ryhB-cm]). RyhB expression was induced by adding 0.6g/L l-arabinose (Wu et al., 2017).

### NADH/NAD^+^ Assay

The intracellular NADH/NAD^+^ ratio was determined using a method described in a previous study (Yang et al., 2015). The steady-state cells were harvested by centrifuging at 12,000rpm, 4°C for 10min. A colorimetric NAD/NADH Assay Kit (ab65348; Abcam, United Kingdom) was used to quantify the NADH/NAD^+^ in cell extracts.

### Analytical Methods

Cell density was assessed by measuring the optical density of the cultures at 600nm (OD_600_) using a UV-721G spectrophotometer. The dry cell weight (DCW) of *E. aerogenes* was subsequently calculated using the empirical formula: DCW (mg/ml)=0.132×OD_600_ (Zhao et al., 2015). Analytes in the gas space, including 2,3-BD and ethanol, were measured using a GC-2010 Plus gas chromatography apparatus (Shimadzu, Japan) equipped with a thermal conductivity detector at 40°C and a Parapak Q column at 200°C, with He as the carrier gas.

Samples comprising 10ml of the fermentation cultures were centrifuged at 10,000× *g* and 4°C for 15min, and the supernatant was then harvested for analysis of metabolites and residual sugar. The concentrations of formate, acetate, lactate, succinate, citrate, acetoin, and ethanol in the culture supernatant were determined by HPLC (LC-20A; Shimadzu, Japan), using a PREP-ODS (H) KIT C-18 column (Shimadzu) kept at 33°C, and a refractive index detector (RID-10A, Shimadzu). The mobile phase was composed of 0.2% aqueous phosphoric acid at a flow rate of 0.8ml/min. Formate, ethanol, lactate, acetate, citric acid, and 2,3-BD had retention times of 4.42, 5.12, 5.38, 5.67, 6.49, and 11.51min, respectively.

## Results and Discussion

### Effects of Knocking Out the *nuoC*, *nuoD*, and *ldh* Genes on 2,3-BD Production

To examine the relationship between NADH dehydrogenase activity, 2,3-BD synthesis, and carbon flux distribution, CRISPR/Cas9 precise editing technology (Wu et al., 2017) was used to delete the *nuoC* and *nuoD* genes, encoding the two subunits of NADH dehydrogenase, resulting in the *E. aerogenes* IAM1183 derivative Ea-1 (Table 1). To increase the production of 2,3-BD, the *ldh* gene was further deleted using λ-Red recombination to limit lactate production in Ea-1, resulting in strain Ea-2 (Table 1). To study the effects of the mutations on metabolite production and carbon source consumption, the strains were cultured in medium containing 40g/L of glucose.

**Figure 1:**
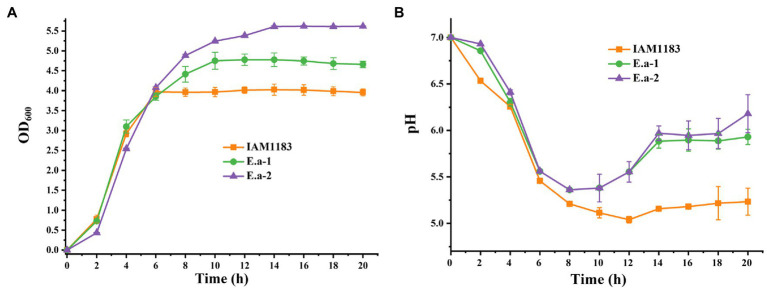
Time-profiles of biomass accumulation (OD_600_) and culture pH for IAM1183 and its mutants. **(A)** Biomass of IAM1183, Ea-1 and Ea-2. **(B)** Culture pH of IAM1183, Ea-1 and Ea-2 (*n*=3).

To measure 2,3-BD production, all strains were cultivated under aerobic conditions in 250-ml shake-flasks with 50ml of glucose medium. The parental strain IAM1183 served as the control. After 20h of cultivation, the cell density of both Ea-1 and Ea-2 was greater than that of IAM1183 (Figure 1A). Compared with the exponential phase of wild-type IAM1183, which lasted 6h, the constructed strains Ea-1 and Ea-2 showed corresponding phases that were ~4 and ~8h longer, respectively (Figure 1A). The pH of the culture broth of Ea-1 and Ea-2 decreased during 20h of batch fermentation at a lower rate than that of IAM1183 (Figure 1B). These results demonstrated that the deletion of *nuoC*, *nuoD*, and *ldh* reduced the acidification rate of Ea-1 and Ea-2, resulting in a higher plateau phase of the mutants (Figure 1). NADH availability and its reduction potential (the intracellular reduced NADH pool) has a stronger effect on the overall yield of 2,3-BD (Yang et al., 2015). Ji et al. (2010) constructed a *Klebsiella oxytoca* strain that does not accumulate 2,3-BD by deleting the aldehyde dehydrogenase gene *aldA*.

In this study, the 2,3-BD titer of Ea-1 reached 70.64mM within 20h of cultivation, which was much higher than the 45.72mM of the wild type (Table 2), indicating that the removal of NADH dehydrogenase significantly improved 2,3-BD production in *E. aerogenes* IAM1138. As shown in Table 2, the double-knockout strain Ea-2 was obviously a superior 2,3-BD producer, with a 84.6% higher product titer than that of the wild-type IAM1183. Furthermore, the 2,3-BD yield from glucose improved 61% compared with the wild-type strain (Table 2). Additionally, the increased growth rate of the Ea-2 mutant also led to an increase in 2,3-butanediol production. It was reported that when LDH is eliminated, the increased NADH availability and redistribution of carbon fluxes lead to an increase in 2,3-BD production (Jung et al., 2012). Metabolically engineered microbes often have lower growth rates than the wild type. The increased microbial growth of the double mutants in this study might be the result of reduced acidification of the medium. Guo et al. (2020) constructed a mutant *K. pneumoniae* strain by deleting three genes, *ldhA*, *adhE* (encoding alcohol dehydrogenase), and *pta* (encoding phosphotransacetylase), resulting in significantly increase the yield of 2,3-BD. Currently, undesirable byproducts (such as lactate, ethanol, and acetate) are also produced during the 2,3-BD fermentation process, which shunts the carbon flow and dramatically decreases the efficiency of 2,3-BD synthesis. Thus, blocking pathways that competitively consume NADH to redistribute carbon flux will be more conducive to the 2,3-BD synthesis.

**Table 2:** Comparison of wild-type *Enterobacter aerogenes* IAM1183 and the mutants Ea-1 to -4 in 20-h shake-flask fermentations using 40g/L of glucose as the carbon source (*n*=3).

End products	Strains				
	IAM1183	Ea-1	Ea-2	Ea-3	Ea-4
Consumed carbon source (mM)	76.93±0.43	75.43±0.77	88.07±0.97	71.99±0.85	76.12±0.36
2,3-Butanediol (mM)	45.72±3.82	70.64±0.29	84.38±2.71	57.73±6.23	72.10±3.94
Formate (mM)	6.03±1.17	4.94±0.53	2.52±0.30	3.19±0.63	1.62±0.99
Acetoin (mM)	45.25±1.76	21.58±1.94	38.57±2.03	31.93±0.77	36.05±1.79
Lactate (mM)	3.21±0.21	4.14±0.41	0.34±0.008	7.17±0.88	0.92±0.33
Ethanol (mM)	6.27±0.77	9.29±0.53	13.95±0.36	6.37±0.63	14.55±1.76
Acetate (mM)	9.14±0.62	6.18±1.03	14.32±1.62	23.71±1.19	17.75±1.54
Citric acid (mM)	1.51±0.08	3.29±0.13	1.16±0.003	0.33±0.08	2.10±0.30
2,3-Butanediol yield (mol/mol Glc)	0.59±0.09	0.93±0.03	0.95±0.02	0.80±0.13	0.94±0.01

### Effect of Overexpressing the sRNA RyhB on 2,3-BD Production

**Figure 2:**
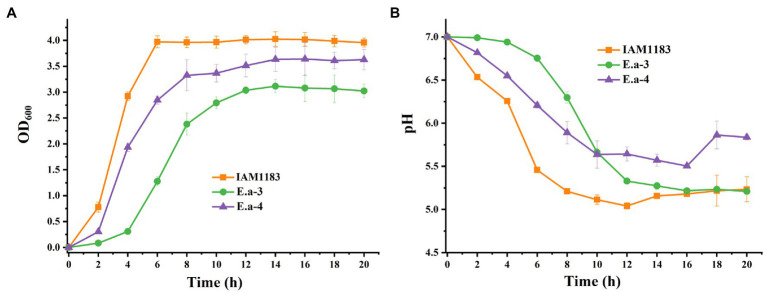
Time-profiles of biomass accumulation (OD_600_) and culture pH for IAM1183 and its mutants. **(A)** Biomass of IAM1183, Ea-3 and Ea-4. **(B)** Culture pH of IAM1183, Ea-3 and Ea-4 (*n*=3).

The plasmid pKK102-ryhB-cm, which overexpresses the small RNA *RyhB*, was introduced into Ea-1 and Ea-2 to generate Ea-3 and Ea-4, respectively. Based on the OD_600_, the final cell densities of Ea-3 and Ea-4 were lower than that of *E. aerogenes* IAM1183 by 13 and 24%, respectively (Figure 2A). Moreover, the two mutants Ea-3 and Ea-4 entered the plateau phase ~4–6h later than the wild-type strain IAM1183, which reached the plateau at 6h (Figure 2A). During 20h of shake-flask cultivation, the pH of the Ea-3 and Ea-4 cultures decreased more slowly than that of IAM1183 (Figure 2B). However, the final pH of the Ea-3 and Ea-4 cultures was lower than that of strains Ea-1 and Ea-2, respectively, implying that overexpressing the sRNA RyhB had an effect on acid production, which inhibited the growth of the strain (Figure 2).

**Figure 3:**
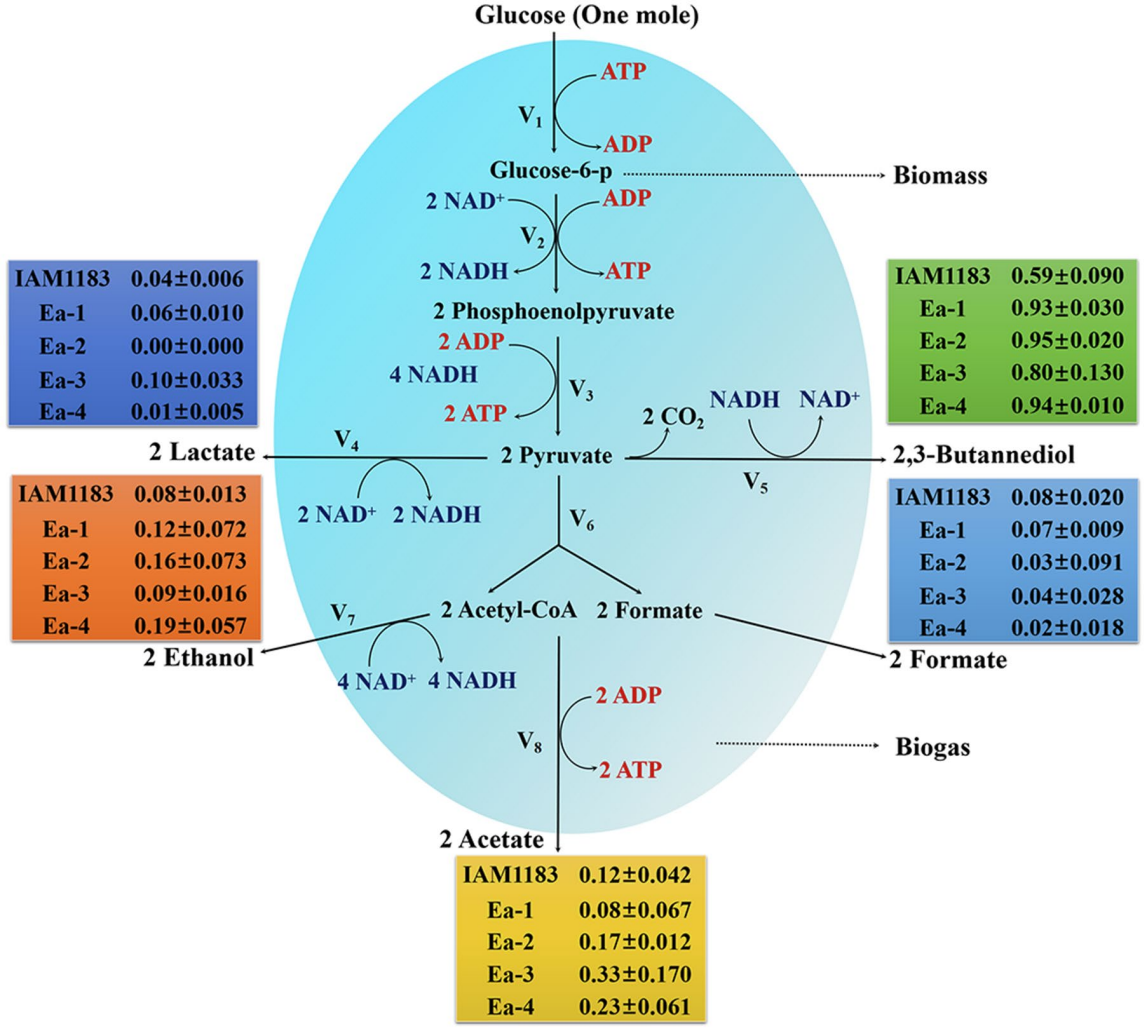
Metabolite profiles of IAM1183 and the mutants Ea-1 to -4 after 20h of shake-flask cultivation with glucose as carbon source (*n*=3). The data shown in Table 2 were used for the calculations.

We also found that overexpression of *RyhB* caused significant decrease in 2,3-BD production (18.3%) in Ea-3 compared with Ea-1, and a modest decrease in Ea-4 (14.6%) compared with Ea-2 when grown on glucose (Table 2; Figure 3). A previous study demonstrated that overexpression of *RyhB* could improve 2,3-BD production under anaerobic conditions, while we found that *RyhB* had no effect on aerobic 2,3-BD production (Wu et al., 2017). In *E. coli*, formate dehydrogenase is downregulated by *RyhB* under aerobic conditions (Massé et al., 2005). Indeed, the formate production of Ea-3 and Ea-4 was significantly lower than that of Ea-1 and Ea-2 (Table 2; Figure 3). Among the by-products, Ea-3 produced 7.17mM lactate and 23.71mM acetate, representing 39.5 and 79.9% increases compared to Ea-1, respectively. Furthermore, Ea-4 produced more acetate and lactate than Ea-2 (Table 2; Figure 3). The resulting acidification of the Ea-3 and Ea-4 cultures may explain their lower 2,3-BD titer. Interestingly, the production of acetate by Ea-3 was increased 2.67-fold compared with Ea-1, while Ea-4 produced 27% more acetate than Ea-2. Similarly, Kang et al. (2012) reported that overexpression of *RyhB* led to a threefold increase in acetate accumulation in *E. coli*, which indicated that *RyhB* had a comprehensive influence on the central glucose metabolism of *E. aerogenes*.

### Effects of Different Carbon Sources on the Mutants

**Figure 4:**
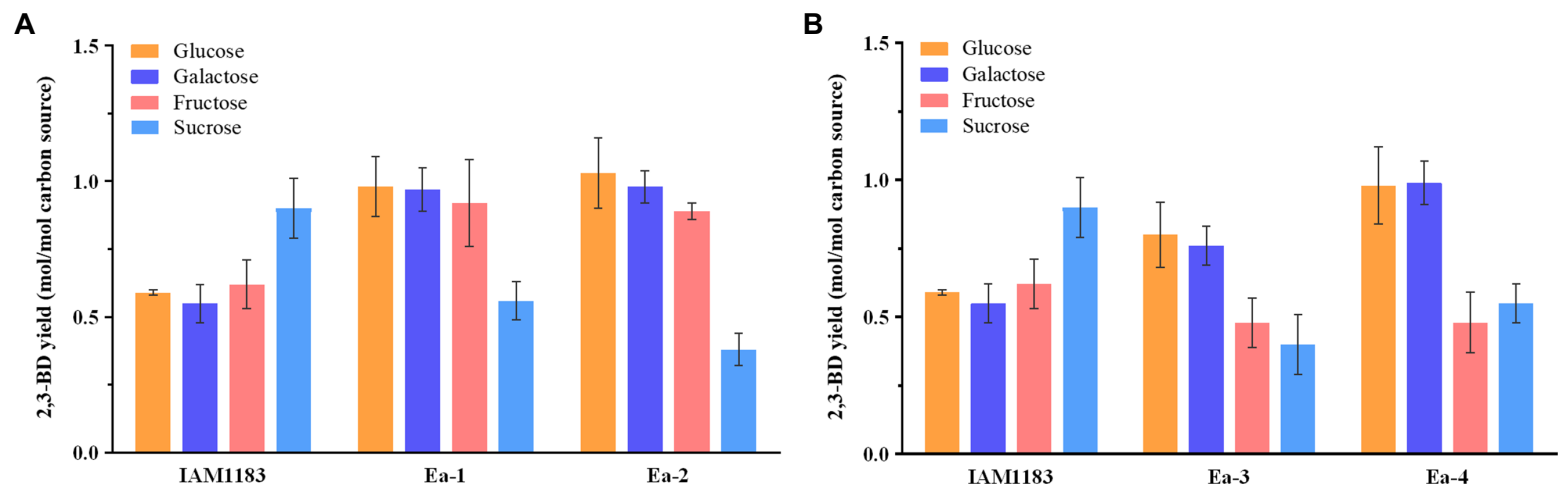
Comparison of 2,3-BD yields from different carbon sources for the wild-type strain and the mutants. **(A)** 2,3-BD yield of IAM1183, Ea-1, and Ea-2. **(B)** 2,3-BD yield of IAM1183, Ea-3, and Ea-4 (*n*=3). The data shown in Tables 2 and 3 were used for the calculations.

*Enterobacter aerogenes* can effectively utilize many different carbon sources (Perego et al., 2000). To detect the effects of different carbon sources on the mutants, shake-flask fermentations were conducted using 40g/L of glucose, fructose, sucrose or galactose (sections “Effects of Knocking Out the nuoC, nuoD, and ldh Genes on 2,3-BD Production” and “Effect of Overexpressing the sRNA RyhB on 2,3-BD Production”) With galactose and fructose, the production of 2,3-BD by Ea-1 was significantly increased, while the carbon source consumption rate was modestly decreased compared with IAM1183 (Table 3; Figure 4A), which was presumably caused by the lower NADH dehydrogenase activity in the two constructed strains, which conserved energy during the exponential growth phase. The concentrations of other fermentation byproducts, such as formate and acetate, all decreased >20% in Ea-1. However, there was a significant increase in lactate production, which, respectively, increased 28.9, 41.8, and 79.6% compared with IAM1183 with all carbon sources except for sucrose (Table 3), which was detrimental for the productivity and yield of 2,3-butanediol (Figure 4A).

**Table 3:** Comparison of wild-type *E. aerogenes* IAM1183 with the mutants Ea-1 and Ea-2 grown for 20h in shake-flask cultures on different carbon sources (*n*=3).

End products	Strains								
	IAM1183			Ea-1			Ea-2		
Carbon source (40g/L)	Galactose	Fructose	Sucrose	Galactose	Fructose	Sucrose	Galactose	Fructose	Sucrose
Consumed carbon source (mM)	77.22±0.18	79.07±0.19	80.82±0.22	76.81±0.68	78.63±0.81	74.33±0.80	92.06±0.72	84.94±0.95	83.89±0.92
2,3-Butanediol (mM)	42.72±1.57	48.94±2.55	73.00±0.58	74.72±11.80	72.44±0.45	41.51±0.71	88.97±5.41	75.59±0.37	32.10±0.19
Formate (mM)	7.10±0.70	2.45±0.37	5.37±1.46	5.54±1.32	1.62±0.39	6.27±0.31	2.99±0.12	2.14±0.16	3.18±0.47
Acetoin (mM)	31.05±1.61	12.83±0.77	35.08±0.69	18.69±0.36	20.83±0.77	21.46±1.99	31.77±1.62	37.35±0.46	25.29±2.19
Lactate (mM)	2.49±0.66	1.13±0.06	1.98±0.22	3.58±0.90	2.03±0.77	0.93±0.03	0.21±0.002	0.52±0.004	0.16±0.013
Ethanol (mM)	5.31±0.45	7.47±1.11	9.61±0.56	5.08±0.17	6.86±0.47	7.42±0.68	12.72±0.04	9.91±0.31	18.91±0.44
Acetate (mM)	4.88±1.36	2.26±0.08	4.71±1.68	4.63±1.15	6.49±0.42	4.68±0.14	6.29±0.58	8.09±0.35	4.89±0.34
Citric acid (mM)	1.56±0.45	1.21±0.12	1.58±0.46	2.99±0.75	4.35±0.32	2.67±0.07	2.12±0.52	1.43±0.28	1.16±0.26
2,3-Butanediol yield (mol/mol Glc)	0.55±0.07	0.62±0.09	0.90±0.03	0.97±0.01	0.92±0.06	0.56±0.07	0.96±0.04	0.89±0.03	0.38±0.06

When grown on galactose and fructose, the 2,3-BD production and carbon source utilization rate of Ea-2 also increased (Table 3; Figure 4A). Similar to glucose, these carbon sources are metabolized *via* the glycolysis pathway. Due to the likely presence of a secondary LDH or a side activity of an unrelated enzyme, Ea-2 still produced a small amount of lactate. The large decrease in lactate accumulation had positive impacts to 2,3-BD productivity, which, respectively, increased by 17.5 and 4.3% compared with Ea-1 on galactose and fructose (Table 3; Figure 4A). The slower decrease in the culture pH in the double mutant likely led to the increase in productivity (Booth, 1985). The double mutant showed an improvement in the consumption rates of all carbon sources. Notably, obvious ethanol accumulation was observed in the double mutant with sucrose as the carbon source, and this increase was accompanied by a reduced 2,3-BD titer. Glucose directly enters the glycolysis pathway and does not require any additional energy to convert it into a usable form (Öhgren et al., 2006). This explains why glucose was the most efficient carbon source, since sucrose requires an additional enzyme and energy input in order for it to be converted into glucose and processed in glycolysis. These processes may lead to the production of more by-products such as ethanol (Bonestroo et al., 1992).

The effect of overexpressing the sRNA *RyhB* was tested in four cultures with various carbon sources (Table 4). The Ea-3 and Ea-4 strains showed a reduction in carbon source consumption, 2,3-BD production (Figure 4B), and yield with all carbon sources compared with Ea-1 and Ea-2, respectively. The accumulation of acidic by-products such as lactate and acetate reduced the 2,3-BD productivity (Table 4; Figure 4B). *RyhB* was reported to have an even stronger effects on intermediary metabolism than on the TCA cycle enzymes, downregulating many different Fe-S-containing metabolic enzymes (Massé et al., 2005; Beauchene et al., 2015), which may explain the reduction in 2,3-BD production in Ea-3 and Ea-4. sRNAs are important regulators of gene expression and physiology in bacteria. RyhB is an iron-responsive sRNA well characterized in *E. coli* and conserved in other *Enterobacteriaceae* (Lillian et al., 2021). Zhang et al. (2018) have demonstrated that increased ATP levels were observed in the ryhB-knockout mutant of *E. coli*, which indicated that the decrease in ATP level has an impact on the lower 2,3-BD production of ryhB-overexpress mutant in our study.

**Table 4:** Comparison of Ea-3 and Ea-4 mutants in 20-h shake-flask cultivations using different carbon sources (*n*=3).

End products	Strains					
	Ea-3			Ea-4		
Carbon source (40g/L)	Galactose	Fructose	Sucrose	Galactose	Fructose	Sucrose
Consumed carbon source (mM)	81.13±0.94	79.07±0.22	72.33±0.65	82.13±0.57	80.36±0.76	69.89±0.62
2,3-Butanediol (mM)	61.67±2.85	37.99±7.28	29.18±0.32	82.01±2.60	38.67±4.17	38.55±3.26
Formate (mM)	1.69±0.34	2.98±0.13	2.20±0.10	1.82±0.28	4.07±0.31	3.23±0.31
Acetoin (mM)	27.72±0. 39	29.27±0.73	25.59±0.70	30.35±1.74	28.63±0.02	23.67±1.51
Lactate (mM)	8.62±0.46	6.94±0.13	9.27±0.75	1.43±0.12	1.66±0.77	1.15±0.01
Ethanol (mM)	7.88±0.26	3.30±1.11	3.02±0.40	23.16±4.40	4.94±1.11	8.18±1.95
Acetate (mM)	7.54±0.25	6.83±0.17	10.27±1.07	4.52±1.12	7.37±0.31	10.20±0.47
Citric acid (mM)	1.45±0.04	1.64±0.12	1.25±0.02	1.60±0.14	1.54±0.14	1.49±0.08
2,3-Butanediol yield (mol/mol sugar)	0.76±0.07	0.48±0.09	0.40±0.11	0.99±0.08	0.48±0.16	0.55±0.07

Interestingly, we found the 2,3-BD production of all mutants on sucrose was significantly decreased compared with the wild type (Figure 4), which was potentially caused by a redox imbalance due to the disruption of NADH dehydrogenase when sucrose was used as substrate (Jung et al., 2014).

### The Intracellular NADH/NAD^+^ Ratios of Different Strains

**Figure 5:**
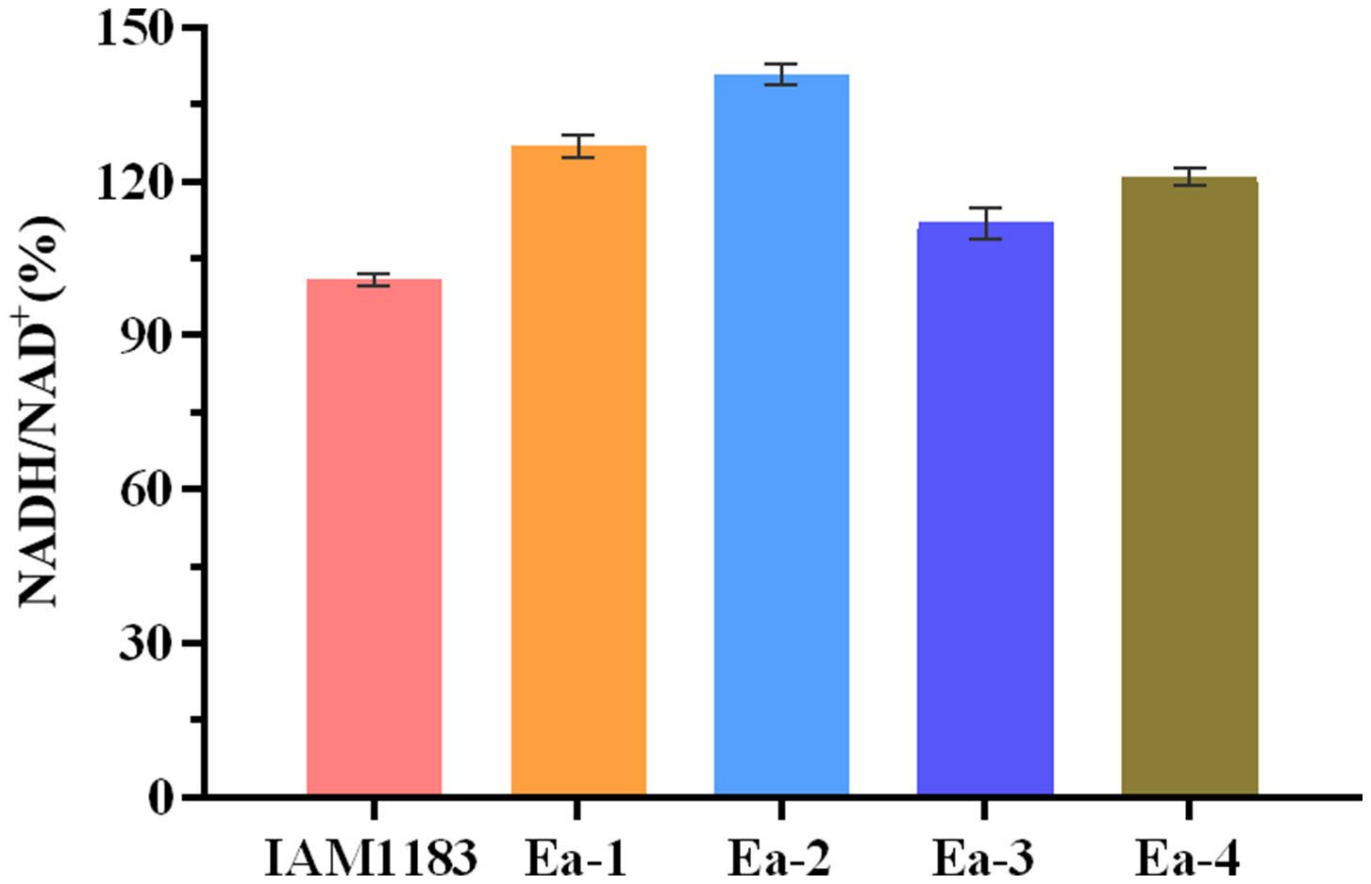
Time profiles of the intracellular NADH/NAD^+^ ratio of the wild-type strain AM1183 and the mutants Ea-1 to -4 (*n*=3). Error bars show the SDs from biological triplicates.

The effects of knocking out *nuoC*, *nuoD*, and *ldhA* on the intracellular NADH/NAD^+^ ratio are shown in Figure 5. After 20h of shake-flask cultivation, the NADH/NAD^+^ ratios of strains Ea-1 and Ea-2, respectively, increased by 25.7 and 39.6% compared with the parental strain. This suggested that the redox balance of strains Ea-1 and Ea-2 was disrupted, which also explained the causes that significantly affect cell growth (Figure 1). As an intrinsic component of the respiratory chain, the mitochondrial NADH: ubiquinone oxidoreductase (complex I) mediates the transmembrane translocation of protons e in the first step of intracellular or mitochondrial NADH oxidation (or alternatively, NAD^+^ reduction; Vinogradov, 2008). The knockout of *nuoC* and *nuoD*, encoding complex I, increased the NADH/NAD^+^ ratio of Ea-1 and redistributed metabolic fluxes to increase 2,3-BD production. Similarly, a previous study showed that deletion of LDH increased NADH availability (Jung et al., 2012). In this study, the molar yield of 2,3-BD and NADH/NAD^+^ ratio of Ea-2 was, respectively, 23 and 11% higher than in Ea-1 (Figure 5; Table 2). This suggests that the deletion of *ldh* also made additional NADH available for other pathways, especially the biosynthesis of 2,3-BD. The strain Ea-3 and Ea-4 showed significant downregulation in NADH/NAD^+^ ratio when compared with the Ea-1 and Ea-2, respectively (Figure 5), which suggested that sRNA RyhB can significantly affect cell metabolism in addition to its role as a regulator of gene expression. Interestingly, a decrease in NADH/NAD^+^ ratios can be observed upon ryhB activation in *E. coli* (Lyu et al., 2019). Thus, RyhB is not merely a genetic-level regulator, its function can also have profound impacts on cell metabolism.

Calderón et al. (2013) reported that the NADH/NAD^+^ ratio was higher in a *ryhB*-deletion strain than in the parental wild type-strain *Salmonella typhimurium*. We found that the NADH/NAD^+^ ratios of Ea-3 and Ea-4 were, respectively, decreased by 11.8 and 14.2% relative to Ea-1 and Ea-2 (Figure 5). These results support a role of *RyhB* in regulating the NADH/NAD^+^ ratio, suggesting that the redox balance of the *E. aerogenes* mutants was disturbed. A similar observation was made in *E. coli* by Lyu et al. (2019), which explains why *RyhB* can significantly affect cell metabolism in addition to its negative effect on 2,3-BD production. Since RyhB acts as a global regulator of glucose metabolism (Kang et al., 2012), it stands to reason that since several of the targets of *RyhB* encode proteins of the TCA cycle, the overexpression of *RyhB* could result in lower levels of NADH in *E. aerogenes*.

### 2,3-BD Production by Ea-2 in a Fermenter

**Figure 6:**
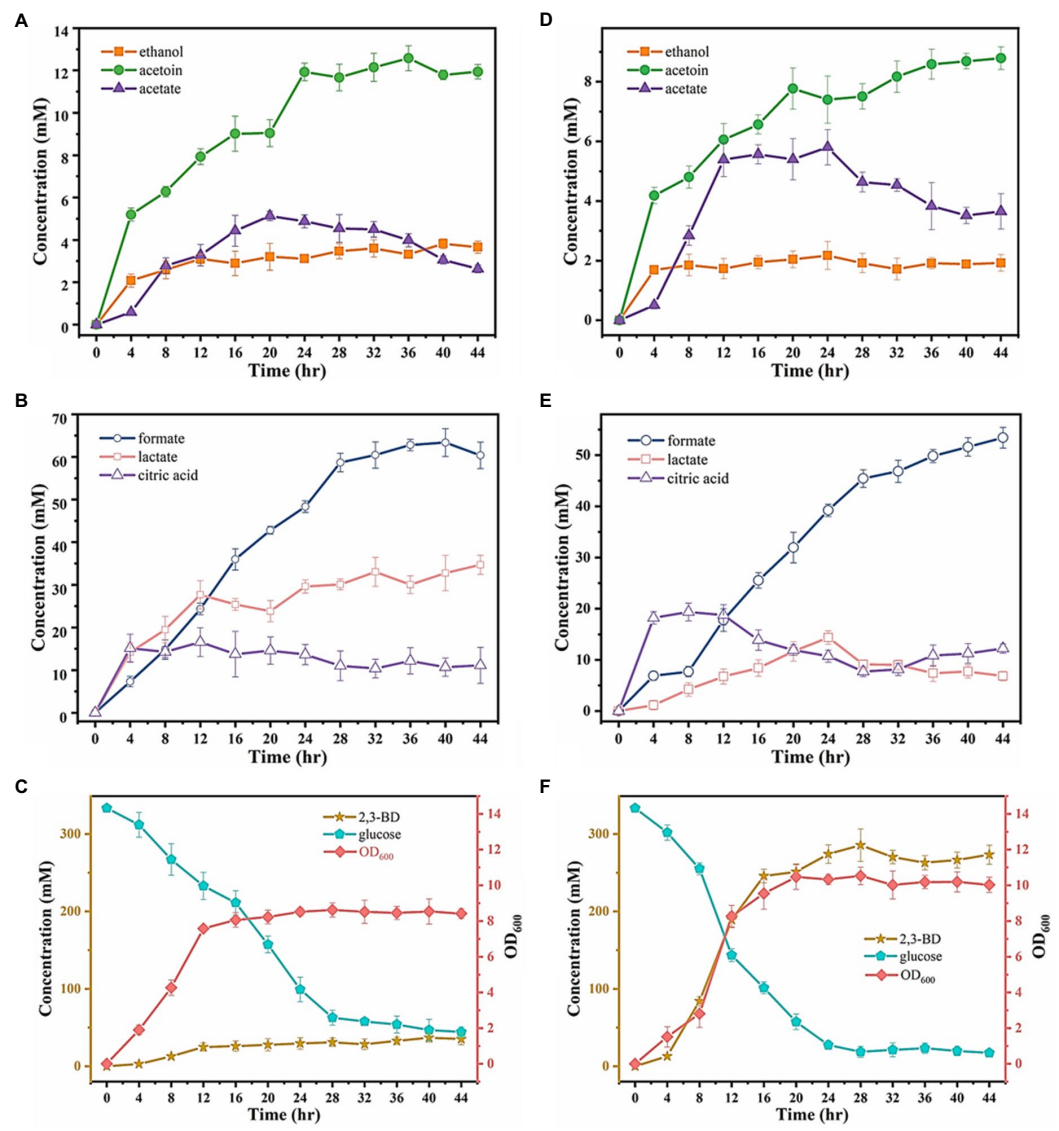
Substrate consumption and produced metabolites after 44h of fermentation for the wild-type strain IAM1183 and the best mutant Ea-2 in a 5-L fermenter. **(A)** Metabolite concentrations of acetate, acetoin, and ethanol in the culture supernatant IAM1183. **(B)** Metabolite concentrations of formate, lactate, and citric acid in the culture supernatant of IAM1183. **(C)** OD_600_, glucose consumption, and concentration of 2,3-BD in the culture supernatant of IAM1183. **(D)** Metabolite concentrations of acetate, acetoin, and ethanol in the culture supernatant of Ea-2. **(E)** Metabolite concentrations of formate, lactate, and citric acid in the culture supernatant of Ea-2. **(F)** OD_600_, glucose consumption, and concentration of 2,3-BD in the culture supernatant of Ea-2. Symbols represent acetoin (green circles), acetate (dark purple triangles), ethanol (orange squares), formate (blue circles), lactate (pink squares), citric acid (light purple triangles), optical density (pink diamonds), consumed glucose (blue pentagons), 2,3-BD (orange stars).

Ea-2 exhibited the highest 2,3-BD productivity in shake-flask fermentations among all the tested strains, with a yield from glucose reaching 1.01mol/mol (Figure 3). We therefore used Ea-2 for a scale-up experiment in a 5-L fermenter with 3L of glucose-containing medium. Ea-2 displayed a more pronounced exponential phase (4h longer than in IAM1183), reaching a 40% higher cell density after cultivation for 44h (Figures 6C,F). More glucose was consumed by Ea-2 during the fermentation, which resulted in an almost 7-fold higher 2,3-BD titer compared with the wild type (Figures 6C,F). There was also an increase in acetate production (Figures 6A,D), while the lactate accumulation rate was clearly reduced in Ea-2 (Figures 6B,E).

The final titers of the major byproducts after 44h of fermentation in the 5-L bioreactor were measured for both Ea-2 and WT, as summarized in Table 5. The WT exhibited higher final concentrations of formate and lactate, while the concentration of ethanol decreased. This was in agreement with the shake-flask fermentations. The mutants generated in this work offer a solid basis for further studies on the fermentative metabolism of *E. aerogenes*, with great application potential for industrial 2,3-BD production.

**Table 5:** Analysis of consumed substrate and anaerobic metabolites after 44h of cultivation of wild-type *E. aerogenes* IAM1183 and Ea-2 in 5-L fermenter.

End products	Strains			
	IAM1183	Ea-2	Change trend	Up or down (%)
Glucose consumption (%)	86.74	94.11	↑	8.49
Residual sugar (mM)	44.21	19.75	↓	55.33
Lactate (mM)	34.70	6.89	↓	80.14
Acetate (mM)	2.63	3.65	↑	38.78
Formate (mM)	60.36	53.63	↓	11.15
Ethanol (mM)	3.66	1.96	↓	46.45
Citric acid (mM)	11.12	12.31	↑	10.7
Acetoin (mM)	11.94	8.79	↓	26.38
2,3-Butanediol (mM)	35.04	252.06	↑	619.35

## Conclusion

**Figure 7:**
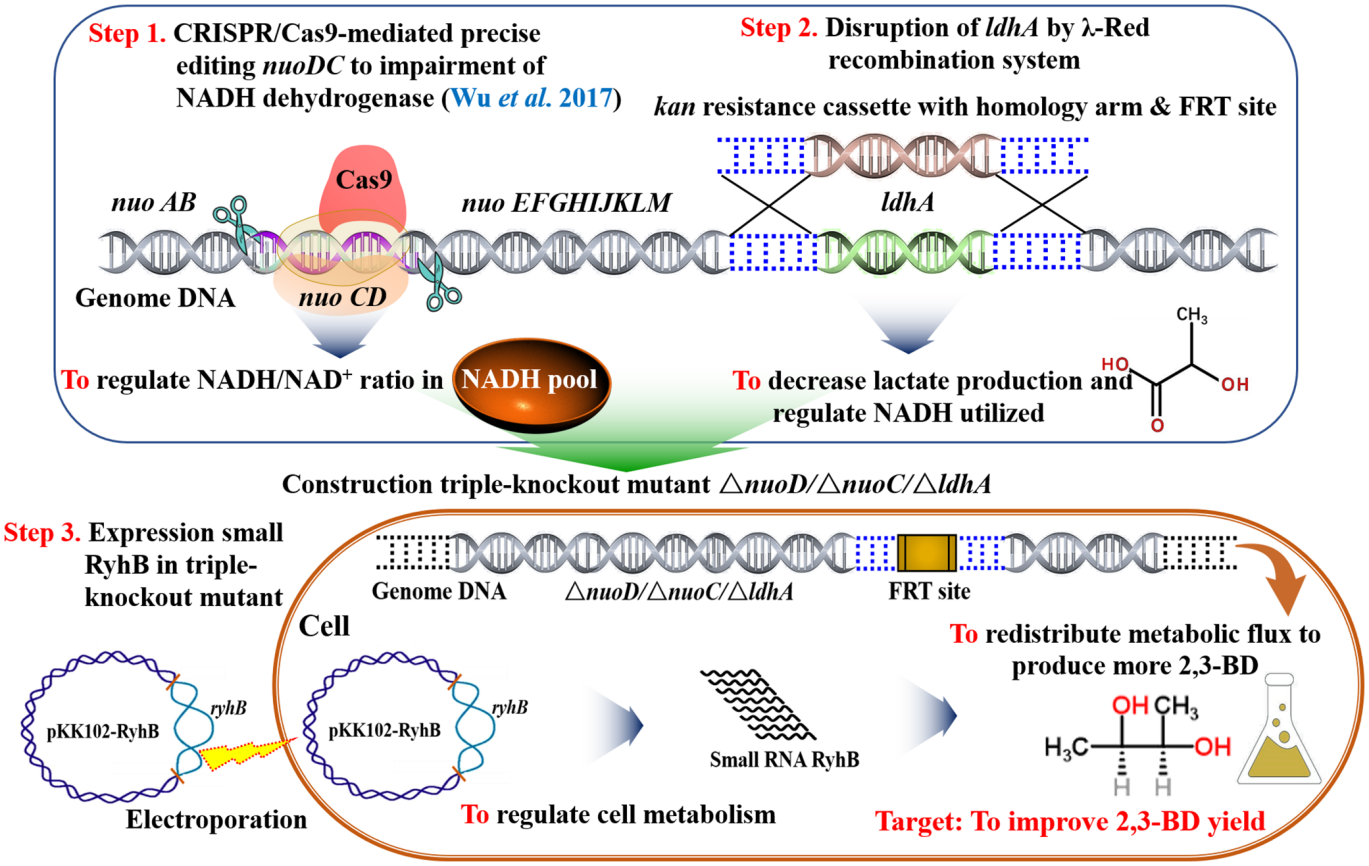
Diagram of primary aerobic metabolic pathways for bacterial 2,3-BD production. Shown are three putative strategies to enhance 2,3-BD yield as outlined in this study.

There are three metabolic pathways for the production of 2,3-BD in *E. aerogenes* (Figure 7). The present work demonstrates that the deletion of NADH dehydrogenase (*ΔnuoC*/*DnuoD* strain Ea-1), alone or in combination with the deletion of LDH (*ΔnuoC*/*DnuoD*/*Dldh* strain Ea-2), can significantly improve the 2,3-BD yield of *E. aerogenes* (Figure 7). However, overexpression of the sRNA RyhB reduced the NADH/NAD^+^ ratio and 2,3-BD production (Figure 7). Metabolic flux analysis of the parental strain IAM1183 and mutants grown on four different carbon sources indicated that the impairment of dehydrogenase is beneficial for 2,3-BD production due to a reduction in acidic by-products, and also indicated that RyhB could redistribute metabolic fluxes in *E. aerogenes*. The 2,3-BD titer produced by the mutant strain Ea-2 in a 5-L fermenter exhibited an almost 7-fold improvement over the wild type. Hence, Ea-2 may be a basis for further improvement of 2,3-BD production *via* metabolic engineering, offering hope to finally achieve biotechnological 2,3-BD production on an industrial scale.

## Data Availability Statement

The original contributions presented in the study are included in the article/supplementary material, further inquiries can be directed to the corresponding author.

## Author Contributions

HZ conceived and supervised the research, analyzed the results, and reviewed and edited the manuscript. HZ and YW designed the experiments. YW, QS, WC, and JY performed the investigations. YX, QS, HY, and FX validated the data. YW, WC, YL, PL, and KJ analyzed the results. YW wrote the original draft of the manuscript. WC modified the manuscript. All authors contributed to the article and approved the submitted version.

## Funding

This work was financially supported by the National Science Foundation of China (grant no. 31970038), the Key Program Funds of Science and Technology Development of Liaoning Province of China (grant no. 2019JH2/10200003).

## Conflict of Interest

The authors declare that the research was conducted in the absence of any commercial or financial relationships that could be construed as a potential conflict of interest.

## Publisher’s Note

All claims expressed in this article are solely those of the authors and do not necessarily represent those of their affiliated organizations, or those of the publisher, the editors and the reviewers. Any product that may be evaluated in this article, or claim that may be made by its manufacturer, is not guaranteed or endorsed by the publisher.
